# IL-6 but Not TNFα Levels Are Associated With Time to Pregnancy in Female Rheumatoid Arthritis Patients With a Wish to Conceive

**DOI:** 10.3389/fphar.2020.604866

**Published:** 2020-12-10

**Authors:** Margot Bongenaar, Hieronymus T. W. Smeele, Erik Lubberts, Radboud J. E. M. Dolhain

**Affiliations:** Department of Rheumatology, Erasmus University Medical Center, Rotterdam, Netherlands

**Keywords:** rheumatoid arthritis, interleukin 6, pregnancy, fertility, BDMARDs (biologic agents)

## Abstract

Fertility issues are common amongst women with rheumatoid arthritis (RA). Interleukin 6 (IL-6) and tumor necrosis factor alpha (TNFα), known key players in RA pathogenesis, have been associated with reproductive disorders. This study investigates the role of these cytokines in decreased fertility in women with active RA. Preconception cytokine measurements of 61 patients from the PARA-cohort, a prospective study on RA and pregnancy, were studied in relation to time to pregnancy as a measure for fertility. IL-6 levels were higher in patients with a time to pregnancy longer than 1 year (*p* = 0.016). Survival analysis of patients stratified by high or low serum IL-6 levels, shows a prolonged time to pregnancy in the high IL-6 group (*p* = 0.045). Univariate cox regression analysis of IL-6 in relation to time to pregnancy as well as multivariate cox regression analysis correcting for age, disease activity, nulliparity, NSAID use and prednisone use were performed, with hazards ratios for log transformed IL-6 of 0.68 (95% CI: 0.51–0.93, *p* = 0.015) and 0.66 (95% CI: 0.43–0.99, *p* = 0.044), respectively. For TNFα, no association with time to pregnancy was found. This study shows that high IL-6, but not TNFα, is associated with decreased fertility in women with RA. This finding provides a rationale to therapeutically target the IL-6 pathway in the time period before pregnancy. More research in the form of large cohort studies on drug safety and the effect of bDMARDS on fertility is needed for implementation of treatment strategies directed at fertility issues in women with RA.

## Introduction

During reproductive age, rheumatoid arthritis (RA) is one of the most common chronic diseases. Female patients with RA have more difficulty conceiving a child, reflected by higher subfertility rates, longer time to pregnancy and decreased family size (Smeele and Dolhain, 2019). Subfertility, defined as a time to pregnancy of greater than 12 months, is related to disease activity in RA patients (Brouwer et al., 2015). Therefore, underlying immunological pathogenic factors associated with active disease are thought to be involved. To date, immunologic mechanisms causing subfertility in RA patients remain unresolved. Identifying which factors influence fertility may have implications on how to optimally treat women with RA and a wish to conceive.

Two well-known factors contributing to RA pathogenesis are interleukin 6 (IL-6) and tumor necrosis factor alpha (TNFα) (Yoshida and Tanaka, 2014; Smolen et al., 2016). In normal physiology, IL-6 and TNFα both act as mediators of the acute phase response. In RA, persistent expression of IL-6 contributes to synovial inflammation and damage and is involved in T-cell migration and activation (Tanaka et al., 2014; McInnes and Schett, 2017). Dysregulation of TNFα in RA plays a major role in synovial proliferation and the pro-inflammatory signaling cascade in the joint. Therapies targeting the IL-6 receptor and TNFα are effective treatment options for RA patients (Taylor and Feldmann, 2009; Kang et al., 2019).

Next to their roles in RA pathogenesis, IL-6 and TNFα play a role in reproduction physiology. Dysregulation of IL-6, up or down, may impair fertility and harm pregnancy (Markert et al., 2011). IL-6 has been described to play a role in several inflammatory disorders of the female reproductive organs. For example in endometriosis, serum IL-6 levels are increased in patients and high levels of IL-6 in peritoneal fluid are linked to infertility (Wang F. et al., 2018; Wang X. M. et al., 2018). Likewise, in polycystic ovary syndrome (PCOS), IL-6 levels are higher than in matched controls and a positive correlation between high circulating IL-6 levels and androgen status was described (Peng et al., 2016). Moreover, serum IL-6 levels are higher in women with unexplained infertility (Demir et al., 2009). To a lesser extent, similar associations for TNFα and reproductive disorders are reported: like IL-6, TNFα is increased in endometriosis and PCOS patients (Iwabe et al., 2002; Wu and Ho, 2003; Ebejer and Calleja-Agius, 2013).

The roles of IL-6 and TNFα in subfertility of RA patients are yet unknown. Unraveling the role of these cytokines in subfertile RA patients might open the door to new treatment strategies. Taking above-stated information into account, we hypothesize that high levels of circulating IL-6 and TNFα in women with active RA contribute to subfertility in these patients.

## Methods

### Study Population

This study is embedded in the Pregnancy-induced Amelioration of RA (PARA) study, a prospective nationwide cohort of pregnant women with RA in the Netherlands (2002–2010) that previously has been extensively described in literature (de Man et al., 2008). Patients fulfilling the 1987 American College of Rheumatology criteria for RA (Arnett et al., 1988) with a wish to conceive or who were already pregnant were included. After completion of the PARA study, patients who gave permission to be contacted for future research were approached to participate in a follow-up study. In this follow-up study, patients filled in questionnaires including inquiries on their reproductive history, mode of conception for each pregnancy, visits to a gynecologist, fertility assessments and fertility treatments. These studies were approved by the Erasmus MC medical ethics review board and was executed in compliance with the Helsinki Declaration. All patients were above 18 years of age and gave a written informed consent.

### Data Collection

In the PARA study, home visits were performed before, three times during and three times after pregnancy. The visit before conception took place if the patient had a desire to conceive and she and her partner did not use any contraceptives. If a patient did not conceive within a year after the first visit, another visit took place. At first visit, data on patient and disease characteristics were collected, as well as medical and obstetric history. At each visit, a physical exam was performed and disease activity was scored using the 28-joint disease Activity Score with three variables based on the C-reactive protein (CRP) level (DAS28-CRP) according to literature (Fransen and van Riel, 2009). CRP was determined using the Tina-Quant CRP Immunological Test System (Roche Diagnostics, Almere, The Netherlands).

Additionally, blood samples were collected and serum was stored at −80°C until use. IL-6 and TNFα serum levels were measured using the IMMULITE 1000 (Siemens Healthcare Diagnostics, Breda, The Netherlands). The intra-run/inter-run mean ± variation coefficient was 105.6 ± 4.9%/87.67 ± 6.1% for IL-6 and 105.6 ± 4.9%/89.5 ± 3.8% for TNFα, with a detection threshold of 2.0 pg/ml. All cytokine levels are presented in pg/ml. Cytokine levels represent the same data as previously described by de Steenwinkel et al. (2013). In that study, the effect of circulating maternal cytokines on birth weight of children was studied at several time points during pregnancy. Measurements were performed on serum taken at the preconception visit as an estimate for cytokine levels during early pregnancy. In this paper, preconception cytokine levels were re-analyzed in relation to time to pregnancy. Therefore, the patients studied represent a subgroup of the patients in the PARA-study: a pre-pregnancy serum should be available and the patients should have given birth during the follow-up period of the study.

### Time to Pregnancy Calculation

Time to pregnancy was calculated as described by Brouwer et al. (2015). At the preconception visit, the date the patient first began trying to conceive actively was noted. Time to pregnancy was calculated as the time elapsed between the first attempt to conceive and the first day of the last menstrual period before pregnancy. If the date of the last menstrual period was not known by the patient, this date was calculated by subtracting 280 days (40 weeks) from the due date based on sonographic examination during early pregnancy. Because in several cases the couple succeeded within the first month of trying to conceive, we calculated a fictitious date by adding 28 days to the start of the last menstrual period for all patients to avoid negative time to pregnancy values.

### Statistical Analysis

Descriptive statistics are presented as mean (SD), number (percentage) or median [interquartile range (IQR)]. Characteristics of study population subgroups stratified by time to pregnancy longer or shorter than 1 year were compared using two-sample t tests, Wilcoxon rank-sum test and two-sample tests of proportions as appropriate. Differences between subgroups in levels of either IL-6 or TNFα were analyzed using Wilcoxon rank-sum tests. Next, patients were stratified in either high or low IL-6 or TNFα based on the median. Study population characteristic differences between high and low cytokine level subgroups were analyzed using two-sample t tests, Wilcoxon rank-sum test and two-sample tests of proportions as appropriate. Differences in time to pregnancy between high and low level cytokine categories were studied using Kaplan–Meier survival curves. Significance between curves was determined using a Gehan–Breslow–Wilcoxon test. Furthermore, cox regression analyses were performed. First, univariate cox regression analyses for time to pregnancy and IL-6 or TNFα were performed. This was followed by multivariable cox regression analyses with variables DAS28-CRP and IL-6 or TNFα. Next, multivariable cox regression analyses were executed including variables age, disease activity, nulliparity, non-steroidal anti-inflammatory drug (NSAID) use and prednisone use next to IL-6 or TNFα. These variables were chosen in line with literature (Brouwer et al., 2015). Since in literature some reproduction disorders have been shown to be associated with increased IL-6 and TNFα levels, also a subgroup analysis was performed excluding all patients who had been diagnosed with a disorder leading to decreased fertility. In addition, those patients with a partner with a known male cause for subfertility were excluded. As CRP is strongly associated with IL-6 in literature (Tanaka et al., 2014), it was investigated whether for daily clinical practice IL-6 could be replaced by CRP in the analyses. For this purpose, Spearman’s correlation of IL-6 and CRP was executed and the same cox regression analyses were performed with CRP levels instead of IL-6 levels to investigate if outcomes are similar. Statistical analyses were two-tailed and results were considered significant if *p* < 0.05. All analyses were performed using Stata 15 (StataCorp LLC).

## Results

### Study Population

We included 61 out of 373 RA patients with a wish to conceive from the PARA cohort. These patients had IL-6 and TNFα levels determined at their preconception visit as well as clinical data available. For three patients, IL-6 and TNFα levels were measured at the second preconception visit, when they had been actively trying to conceive for 1 year. The mean age (SD) of the study population was 31.1 (3.5) years, duration of disease (IQR) was 6.0 (1.7–10.9) years and mean disease activity (DAS28-CRP) prior to conception (SD) was 3.76 (0.97). Of all included patients, 31 (51%) were anti-citrullinated peptide antibodies (ACPA) positive, 35 (57%) were rheumatoid factor positive, 34 (56%) had erosive disease, 35 (57%) were nulliparous and 19 (31%) had a time to pregnancy >12 months. The following medication was used: sulfasalazine by 19 (31%) patients, hydroxychloroquine by 2 (3%), prednisone by 26 (43%), azathioprine by 1 (2%) patient, etanercept by 1 (2%) patient and gold therapy by 2 (3%) patients. Subgroup analysis showed higher prednisone use in patients with a time to pregnancy longer than 12 months. Described characteristics can be found in Table 1.

**TABLE 1:** Clinical and demographic features of the complete study population of 61 patients with rheumatoid arthritis and a wish to conceive, and subgroups divided by a time to pregnancy (TTP) shorter or longer than 1 year.

Variable	Study population n = 61	TTP < 1 year n = 42	TTP > 1 year n = 19	*p*
Age in years—mean (SD)	31.1 (3.5)	31.1 (3.5)	31.2 (3.5)	0.913
Duration of disease in years—median (IQR)	6.0 (1.7–10.9)	5.5 (2.0–8.6)	9.0 (1.0–13.2)	0.544
DAS28-CRP—mean (SD)	3.76 (0.97)	3.74 (0.99)	3.80 (0.94)	0.833
ACPA positive—n (%)	31 (51)	19 (45)	12 (63)	0.241
RF positive—n (%)	35 (57)	22 (52)	13 (68)	0.195
Erosive disease—n (%)	34 (56)	22 (52)	12 (63)	0.433
Nulliparity—n (%)	35 (57)	24 (57)	11 (58)	0.956
Medication preconception—n (%)				
None	21 (34)	17 (40)	4 (21)	0.139
NSAIDs	20 (33)	11 (26)	9 (47)	0.103
*Prednisone*	26 (43)	*14 (33)*	*12 (63)*	0.029*
Sulfasalazine	19 (31)	15 (36)	4 (21)	0.252
Hydroxychoroquine	2 (3)	1 (2.4)	1 (5.3)	0.558
Gold therapy	2 (3)	2 (4.8)	0 (0)	0.333
Azathioprine	1 (2)	0 (0)	1 (5.3)	0.134
Etanercept	1 (2)	1 (2.4)	0 (0)	0.498

ACPA, anti-citrullinated peptide antibodies; RF, rheumatoid factor; DAS28-CRP, 28-joint disease Activity Score with three variables based on the C-reactive protein level; NSAIDs, non-steroidal anti-inflammatory drugs. *= p < 0.05

Fourty six out of all 61 (75.4%) patients responded to the invite for follow-up study for investigation of reproduction and fertility. Of this population, eight patients (17.4%) were diagnosed with a reproductive disorder: four patients out of 46 (8.7%) were diagnosed with anovulation, 1 (2.2%) with endometriosis, 1 (2.2%) with early menopause, 1 (2.2%) with blockage of fallopian tubes, 1 (2.2%) patient with a male factor influencing fertility and 1 (2.2%) patient with anovulation as well as a male factor.

### Serum Interleukin 6 and Tumor Necrosis Factor Alpha Levels

In 14 out of 61 (23.0%) observations, IL-6 measurements were below the detection limit. Therefore, for all measurements below the detection limit, the IL-6 level was defined at the threshold value of 2.0 pg/ml. IL-6 medians (IQR) were 3.44 (2.0–6.77) and 11.7 (2.67–21.4) pg/ml for patients with a time to pregnancy <12 months (*n* = 42) and patients with a time to pregnancy >12 months (*n* = 19), respectively. IL-6 levels were significantly higher in patients with a time to pregnancy longer than 1 year (*p* = 0.016).

**FIGURE 1:**
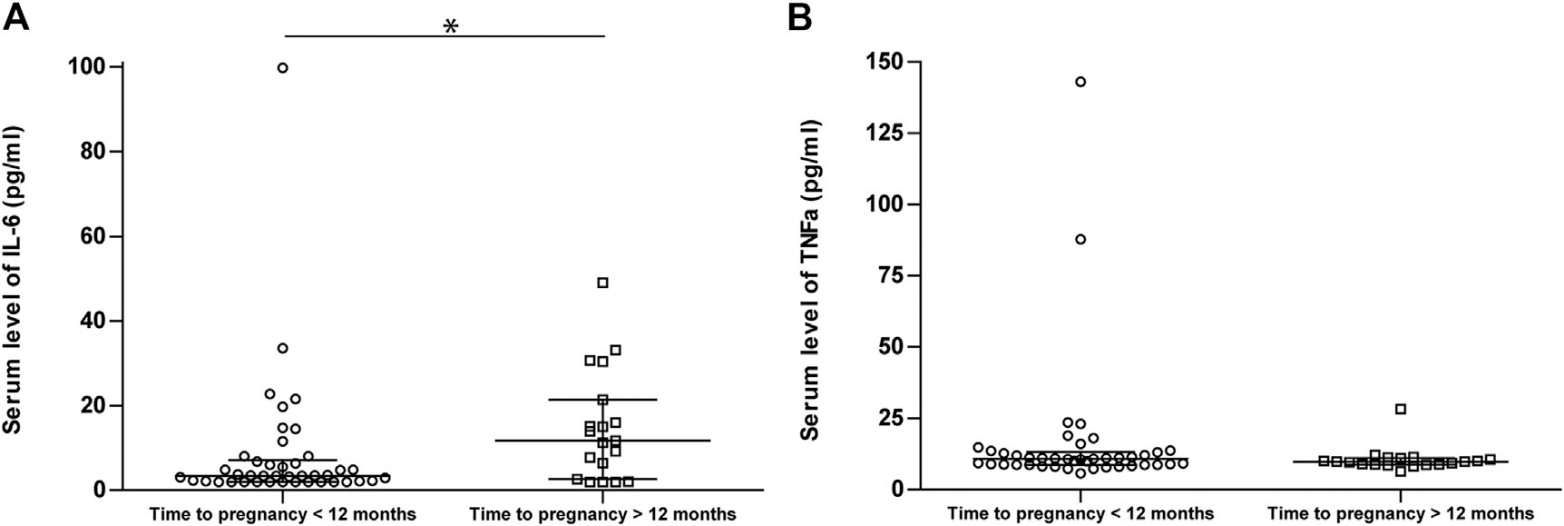
Boxplots showing interleukin 6 (IL-6) and tumor necrosis factor alpha distributions with median and interquartile range stratified by time to pregnancy (**A,B**, respectively). The *y*-axis represents serum cytokine levels of Rheumatoid Arthritis patients with a wish to conceive (pg/ml), the *x*-axis shows patient groups stratified for time to pregnancy. IL-6 levels were statistically significant higher in patients with a time to pregnancy >12 months compared to patients with a time to pregnancy <12 months (*p* = 0.016) **(A)**. * = *p* < 0.05.

For TNFα, all measurements were above the detection limit. TNFα median serum levels (IQR) were 9.76 (8.78–11.1) pg/ml for patients with a time to pregnancy <12 months and 10.75 (8.72–13.0) pg/ml for patients with a time to pregnancy >12 months. There was no significant difference between these groups (*p* = 0.33). Boxplots depicting IL-6 and TNFα distributions categorized by time to pregnancy are shown in Figures 1A,B, respectively.

### Interleukin 6 and Time to Pregnancy

**FIGURE 2:**
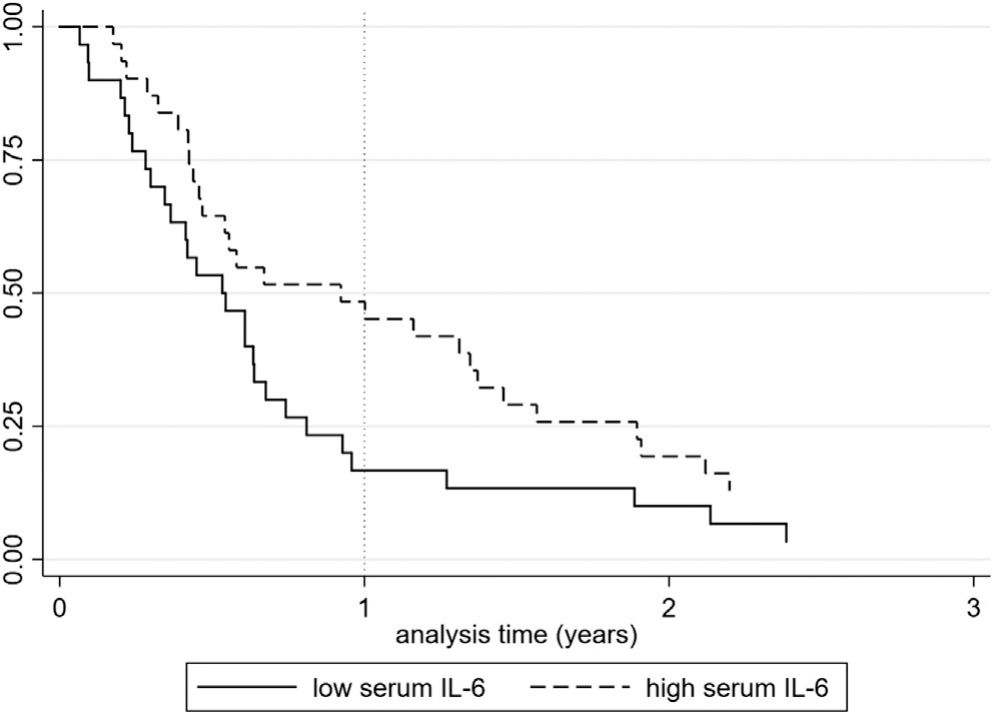
Kaplan-Meier survival curves showing time to pregnancy in interleukin 6 (IL-6) high (*n* = 31) vs. IL-6 low (n = 30) patients. The *y*‐axis shows the proportion of patients who are not yet pregnant, the *x*‐axis shows the analysis time (years). The survival curve shows that time to pregnancy is longer for patients with high levels of IL-6 (*p* = 0.045).

Patients were stratified in IL-6 low and IL-6 high categories based on the median IL-6 level of the complete study population (median = 4.75 pg/ml). In the IL-6 high subgroup, disease activity (DAS28-CRP) was higher, patients used more NSAIDs and medication use in general was more frequent than in the IL-6 low group (*p* = 0.0005, *p* = 0.0364 and *p* = 0.0022, respectively). Kaplan–Meier survival analysis (*n* = 61) shows a different time to pregnancy between the groups with either high or low IL-6. The group with high IL-6 shows a prolonged time to pregnancy compared to the group with low levels of serum IL-6 (*p* = 0.045). This survival curve is shown in Figure 2.

IL-6 levels were logarithmically transformed before being imputed in the regression analysis. Univariate cox regression analysis showed that IL-6 is associated with time to pregnancy: hazard ratio (HR) 0.68 (95% CI: 0.51–0.93, *p* = 0.015). Cox regression analysis with the variables IL-6 and DAS28-CRP showed that higher IL-6 scores were associated with a reduced chance of pregnancy, corrected for disease activity: HR for IL-6 0.66 (95% CI: 0.46–0.94, *p* = 0.022). The multivariate cox regression, with variables IL-6, age, disease activity, nulliparity, NSAID use and prednisone use showed a significant association between IL-6 and time to pregnancy with a HR 0.66 (95% CI: 0.43–0.99, *p* = 0.044).

Furthermore, using the data from the follow-up study (*n* = 46), all patients with a diagnosis of a reproductive disorder that could explain decreased fertility (*n* = 8) were excluded in a subgroup analysis. Seven out of eight exclusions belonged to the subfertile group with a time to pregnancy longer than 12 months. Their median (IQR) IL-6 level was 7.86 (1.34–27.35) pg/ml. Univariate analysis of IL-6 (*n* = 38) showed a significant association of IL-6 levels with time to pregnancy: HR 0.69 (95% CI: 0.48–0.99, *p* = 0.046). Next, multivariate analysis with variables IL-6, age, disease activity, nulliparity, NSAID use and prednisone in relation to time to pregnancy was performed on the same subgroup (*n* = 38) and showed a significant association between IL-6 and time to pregnancy: HR 0.54 (95% CI: 0.32–0.92, *p* = 0.023).

### Tumor Necrosis Factor Alpha and Time to Pregnancy

After stratification of patients in TNFα low and high categories by the median of 9.76 pg/ml, patients in the TNFα high subgroup had longer duration of disease (*p* = 0.0182). Survival analysis for TNFα stratified by high and low serum levels did not show the same pattern as IL-6. Kaplan Meier survival (*n* = 61) showed that having high or low serum TNFα did not have an effect on time to pregnancy (*p* = 0.62). Levels of TNFα were logarithmically transformed before being imputed into the models. Univariate cox regression analysis showed that TNFα is not associated with time to pregnancy: HR 1.41 (95% CI: 0.88–2.24, *p* = 0.15). Cox regression analysis with the variables TNFα and DAS28-CRP and analysis with variables TNFα, age, disease activity, nulliparity, NSAID use and prednisone use did not show an effect of TNFα on the probability of pregnancy: HR 1.41 (95% CI: 0.90–2.25, *p* = 0.14) and HR 1.34 (95% CI: 0.75–2.39, *p* = 0.33), respectively. Univariate analysis of the subgroup excluding all patients with a reproductive disorder or a male factor [median (IQR) TNFα level of excluded patients: 8.62 (8.10–9.29) pg/ml] that could explain decreased fertility (*n* = 38) showed no association of TNFα with time to pregnancy: HR 1.12 (95% CI: 0.63–1.98, *p* = 0.69). Lastly, multivariate analysis on the same subgroup including TNFα, age, disease activity, nulliparity, NSAID use and prednisone in relation to time to pregnancy did not show an association between TNFα levels and time to pregnancy: HR 1.41 (95% CI: 0.71–2.78, *p* = 0.31).

### C-Reactive Protein and Time to Pregnancy

IL-6 and CRP levels are strongly correlated in our study population (Spearman’s rank correlation, *r*_*s*_ = 0.71). CRP values were log transformed for cox regression analyses. Univariate analysis of CRP in relation to time to pregnancy showed a significant effect on time to pregnancy: HR 0.81 (95% CI: 0.65–1.00, *p* = 0.048). Multivariate cox regression analysis with the variables CRP and DAS28-CRP and analysis with variables CRP, age, disease activity, nulliparity, NSAID use and prednisone use both showed an association between CRP levels and time to pregnancy: HR 0.76 (95% CI: 0.59–0.99, *p* = 0.044) and HR 0.76 (95% CI: 0.59–0.98, *p* = 0.036), respectively. No significant association between CRP and time to pregnancy was found in the subgroup analyses excluding patients with a reproductive disorder or a male factor that could explain decreased fertility (*n* = 38) (univariate analysis HR 0.83 (95% CI: 0.64–1.06, *p* = 0.137); multivariate analysis with variables CRP, age, disease activity, nulliparity, NSAID use and prednisone use HR 0.74 (95% CI: 0.53–1.03, *p* = 0.075).

## Discussion

Our study is the first to evaluate IL-6 and TNFα as potential influencing factors of subfertility in RA patients. We showed that IL-6 is increased in RA patients with a time to pregnancy longer than 12 months. High IL-6 levels are associated with a prolonged time to pregnancy, even when corrected for potential confounders, like disease activity, medication and diagnosis of reproductive disorders, in a large cohort of RA patients with a wish to conceive. For TNFα levels, no association with fertility was found.

This report, combined with previous studies on the link between IL-6 and reproductive disorders and fertility (Demir et al., 2009; Peng et al., 2016; Wang F. et al., 2018; Wang X. M. et al., 2018), provides an indication that high levels of circulating IL-6 hamper fertility. However, the biological mechanisms behind this phenomenon are not known. A possible explanation could be the role of IL-6 in T cell differentiation. IL-6 is a known influencer thereof, driving follicular helper T cell differentiation and enabling T helper 17 cell over regulatory T cell differentiation (Schinnerling et al., 2017). A disturbed T helper 17 and regulatory T cell balance has been associated with fertility and pregnancy disorders through lack of immunologic tolerance, which is pivotal for implantation and pregnancy maintenance (Jasper et al., 2006; Figueiredo and Schumacher, 2016).

Despite the association between IL-6 and fertility, IL-6 might not directly be involved in subfertility pathogenesis. It is possible IL-6 is a proxy for something else, such as a disease related factor. For example, IL-6 levels are known to be associated with disease activity (Madhok et al., 1993). Therefore, in our analyses, a correction was made for disease activity score and other possible confounders based on literature. Another possibility is that IL-6 could be a proxy for other factors in the same inflammatory cascade. Fertility immunology is an upcoming field and exact mechanisms surrounding conception and implantation, certainly within RA patients, are yet to be resolved. More research on fertility biology is needed to elucidate the exact role of IL-6 as well as T helper subset balance in this matter.

Within the subgroup with a time to pregnancy shorter than 1 year, some patients with high IL-6 levels are present (Figure 1A). However, since there is only one cytokine measurement available representing the complete preconception time period, it is unclear if cytokines levels were stable in these patients. A possible explanation for these outliers, next to chance, is temporarily high IL-6 levels in the context of infection.

Since decreased fertility is an important issue in women with RA during reproductive age, treatment of patients with a wish to conceive should not only target the immune system in a way that controls maternal disease activity, but also take into account how therapy could affect fertility. Based on this study, the IL-6 pathway might be considered as a therapeutic target in patients with RA and a wish to conceive. Therapies directed at the IL-6 receptor, such as tocilizumab, could be a promising option for those patients. However, for these therapeutic agents, little is known about drug safety during pregnancy. Due to lack of safety data, current guidelines state that tocilizumab should be stopped in RA patients with a wish to conceive (Götestam Skorpen et al., 2016). Small studies report no adverse pregnancy outcomes in patients exposed to tocilizumab, but numbers are too limited to be conclusive on the matter (Nakajima et al., 2016). Consequently, to this date, tocilizumab is not an appropriate therapeutic option for RA patients in the time period before pregnancy, but could be seriously considered in the future, when there is more clarity on teratogenicity.

Meanwhile, TNFα inhibition (TNFi) is a widely used treatment option for RA and current guidelines state that TNFα inhibitors are appropriate therapeutic agents for patients with a wish to conceive (Götestam Skorpen et al., 2016; Sammaritano et al., 2020). Although our analyses show no correlation of circulating TNFα-levels with fertility, TNFα is a driver of IL-6 and use of TNFi has been shown to decrease circulating IL-6 levels (Charles et al., 1999). Moreover, TNFi has been reported to expand regulatory T cells (Nguyen and Ehrenstein, 2016). Therefore, treatment with TNFi could be a potential option to increase fertility in women with active RA. In a small study, it was indeed reported that TNFi decreases time to pregnancy in RA patients with a wish to conceive (Shimada et al., 2019). Potential effects of TNFi on pregnancy outcomes should be considered. Increased risks associated with TNFi of preterm birth, caesarean section and small for gestational age children have been previously reported (Bröms et al., 2020). However, given the study design and the fact that high disease activity is known to negatively influence pregnancy outcomes (Smeele and Dolhain, 2019), these effects might not be drug-derived, but due to confounding by indication.

Given the fact that IL-6 serum levels are correlated to disease activity (Madhok et al., 1993), as also displayed by our results, effective treatment of RA patients with other DMARDs such as sulfasalazine presumably decrease IL-6 levels and possibly enhance fertility as well. More evidence on the effect of different DMARDs on fertility from large cohort studies is needed to confirm this effect and to further elucidate the mechanisms behind it.

In daily clinical practice, IL-6 levels are not routinely measured. This makes the possibility to use IL-6 levels as a potential biomarker for fertility in RA patients with a wish to conceive less practical. However, IL-6 is a strong driver of CRP and high CRP levels are predictive for high IL-6 (Tanaka et al., 2014). For that reason all analyses were performed with CRP instead of IL-6 and for the main analyses similar results were found. Therefore, in daily practice, when no IL-6 levels are available, also CRP is a potential indicator for time to pregnancy in RA-patients with a wish to conceive. Analysis of a larger patient population would give more insight into the value of CRP as a clinical biomarker for fertility.

No association of TNFα levels with fertility was found. This raises the question whether our study is adequately powered to show the potential influence of TNFα on fertility. A post-hoc power calculation shows that, based on the data in this study, over 1,300 patient inclusions are needed to demonstrate a statistically significant difference. This is an indication that a potential difference in TNFα levels is probably not clinically relevant.

Previous reports have shown the PARA cohort to be a representative study population. However, cytokine levels were only determined in samples of patients who had serum from the preconception time point available and who had eventually successfully achieved pregnancy and delivered a child, because of previous research purposes (de Steenwinkel et al., 2013). This led to inclusion of 61 out of 373 total patients in the PARA cohort. Nevertheless, we do not expect that this causes the results to be invalid. Our study population is skewed to a relatively more fertile subgroup of the complete PARA cohort and the results still show an association between IL-6 and time to pregnancy. Although it is presumable that IL-6 levels are high in infertile RA patients as well, this group is not represented in the study population and should be further investigated.

In conclusion, this study shows that high IL-6 levels, but not TNFα, are associated with decreased fertility in women with RA and a wish to conceive. This finding provides a rationale to therapeutically target the IL-6 pathway in the time period before pregnancy.

## Data Availability Statement

The raw data supporting the conclusions of this article will be made available by the authors, without undue reservation.

## Ethics Statement

The studies involving human participants were reviewed and approved by Erasmus MC medical ethics review board. The patients/participants provided their written informed consent to participate in this study.

## Author Contributions

MB and HS drafted the work, analyzed the data and wrote the manuscript. EL and RD critically revised the text and figures. All authors approved the final manuscript for publication.

## Funding

This work was supported by the Dutch Arthritis Foundation (ReumaNederland) (project numbers: LLP-26 and NR 19-2-401) and UCB Pharma. The funders were not involved in the study design, collection, analysis, interpretation of data, the writing of this article or the decision to submit it for publication.

## Conflict of Interest

The authors declare that the research was conducted in the absence of any commercial or financial relationships that could be construed as a potential conflict of interest.
